# Intrinsic Dynamics Analysis of a DNA Octahedron by Elastic Network Model

**DOI:** 10.3390/molecules22010145

**Published:** 2017-01-16

**Authors:** Guang Hu, Lei He, Federico Iacovelli, Mattia Falconi

**Affiliations:** 1 Center for Systems Biology, Soochow University, Suzhou 215006, China; 2 Cambridge-Suda (CAM-SU) Genomic Resource Center, Soochow University, Suzhou 215123, China; lhe@suda.edu.cn; 3 Department of Biology, University of Rome “Tor Vergata”, Rome 00133, Italy; federico.iacovelli@uniroma2.it (F.I.); falconi@uniroma2.it (M.F.)

**Keywords:** DNA nanotechnology, molecular dynamics, hinge regions, collective motions, Gaussian Network Model, Anisotropic Network Model

## Abstract

DNA is a fundamental component of living systems where it plays a crucial role at both functional and structural level. The programmable properties of DNA make it an interesting building block for the construction of nanostructures. However, molecular mechanisms for the arrangement of these well-defined DNA assemblies are not fully understood. In this paper, the intrinsic dynamics of a DNA octahedron has been investigated by using two types of Elastic Network Models (ENMs). The application of ENMs to DNA nanocages include the analysis of the intrinsic flexibilities of DNA double-helices and hinge sites through the calculation of the square fluctuations, as well as the intrinsic collective dynamics in terms of cross-collective map calculation coupled with global motions analysis. The dynamics profiles derived from ENMs have then been evaluated and compared with previous classical molecular dynamics simulation trajectories. The results presented here revealed that ENMs can provide useful insights into the intrinsic dynamics of large DNA nanocages and represent a useful tool in the field of structural DNA nanotechnology.

## 1. Introduction

Owing to its programmable nature, DNA is not only considered a carrier of genetic information but also an ideal prototyping material for the design of well-ordered nanostructures [1,2]. To date, a plethora of DNA nanocages, including tetrahedra [3], cubes [4], octahedra [5], dodecahedra [6], icosahedra [7], Bucky balls [6] and tripods [8] have been self-assembled using different strategies. These DNA nanocages have also been reported to have potential applications in drug delivery, vaccine development and to enhance catalytic activity [9,10,11]. Underlying molecular mechanisms for the formation of these DNA assemblies is still a major challenge in DNA nanotechnology. The experimental production of these complex DNA nanocages is quite expensive, and simulative structural and dynamics studies may greatly help in solving issues involving the assembly.

In the last years, we have been involved in the use of molecular dynamics (MD) simulations of DNA nanocages to characterize their structures and dynamics [12]. In detail, we have investigated local and global structural and mechanical properties of a truncated octahedral DNA nanocage family [13], probed the roles of thymidine linkers in the stability of DNA nanocages [14,15,16,17], and investigated the temperature dependent encapsulation mechanism of a DNA nanostructure [10]. oxDNA, a coarse grain model considering DNA at the nucleotide level, has been proposed to study the self-assembly of DNA nanostructures [18,19,20], mainly to simulate large systems, such as DNA origami based assembly [21]. Mathematical models based on knot and topological graph theory have been built to unravel the basic structural rules driving the assembly of these nanostructures and design the DNA nanocages topologies [22,23,24,25,26]. A computational program called Polygen has also been developed to easily engineer DNA nanocages [27].

Elastic Network Models (ENMs) are widely used coarse-grained methods that can sensitively capture the slow dynamics of biomolecules [28]. In ENM, a biomolecule can be described as particles connected by springs [29] and therefore the intrinsic flexibility and collective motions of the biomolecule are treated as a set of normal modes within an oscillating system. Two main types of ENM were implemented: Gaussian Network Model (GNM) is a simple but effective method to explore intrinsic flexibility [30,31], and Anisotropic Network Model (ANM) is able to describe the large-scale collective motions that are relevant to the function [32,33]. Comparative studies of ENM and MD have pointed out a good correspondence in the description of dynamics profiles between the two methods [34,35,36]. Intrinsic dynamics refers to the conformational changes intrinsically favored by the native topology of three-dimensional (3D) structure, which is often relevant to biological function. ENMs are becoming widely applied methods to study the intrinsic dynamics of biosystems, because of their simplicity, robustness, low computational cost and relatively high accuracy [37,38]. Recently, the ENM has been applied for guiding the molecular design of RNA nanocages [39].

In this paper, the ENM methods have been used to study the structure-encoded dynamical properties (or intrinsic dynamics) of a DNA nanostructure composed by eight oligonucleotides that form a truncated octahedron including 12 B-DNA double helices, constituting the edges of the structure, connected by short single-stranded linkers composing the six vertices (Table S2 and Figure S1). Furthermore, we have tested the utility and extent of ENMs applicability in DNA nanocages comparing with the previous MD result. Finally, the developments, limitations and future directions for describing intrinsic dynamics in DNA nanocages using ENMs have been discussed.

## 2. Results

### 2.1. Comparisons of Fluctuations between the Molecular Dynamics and Elastic Network Models

**Figure 1:**
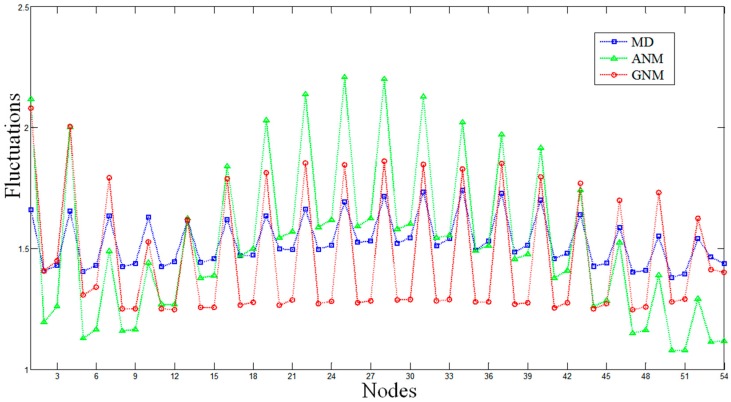
The Root-Mean-Square Fluctuations (RMSF) of the DNA octahedron derived by molecular dynamics (MD, the blue line), Anisotropic Network Model (ANM, the green line), and Gaussian Network Model (GNM, the red line). The RMSFs based on ANM and GNM are calculated when each nucleotide is represented by three nodes. Average fluctuations for only one strand composing the double helices are plotted.

The MD and ENM fluctuations of the DNA octahedron were calculated to evaluate the flexibilities of each DNA strand. The fluctuations from MD have been evaluated by calculating the Root-Mean-Square Fluctuations (RMSF) based on a 50 ns trajectory [27], while that from of ENM is obtained by calculating square fluctuations based on all normal modes. Comparisons of the average fluctuations for each strand predicted by MD, ANM and GNM are shown in Figure 1 and Figures S2 and S3. The RMSFs based on MD and GNM are rescaled so that the average of the fluctuations matches the average of the ANM fluctuation. All profiles show similar overall behaviors, in which almost every peak in the MD data has counterparts, in both the ANM and GNM curves. The lowest fluctuation lies in the central part of a helix, while the highest fluctuation appears on the strand tails, confirming the high conformational variability of the polyhedral vertices represented by single strand bridges connecting the double helices.

It should be noted that each nucleotide can be represented by one (P atom), two (P and O4* atoms) or three nodes (P, C4* and C2 atoms) in both the ANM and the GNM approaches. Figures S2 and S3 show the comparisons between the MD and ENM based on one or two nodes. For the ENMs based on one node, the correlation coefficients between MD and ANM, MD and GNM, ANM and GNM are 0.53, 0.39 and 0.92, respectively. For ENMs based on two nodes, the correlation coefficients between MD and ANM, MD and GNM, ANM and GNM are 0.79, 0.63 and 0.64, respectively. Figure 1 shows the results for the ENMs based on three nodes, which indicate the largest similarity between MD and ENM in the described flexibilities. In this case, the correlation coefficients between MD and ANM, MD and GNM, and ANM and GNM are 0.82, 0.79 and 0.72, respectively. These comparative results indicate that ENM based on three nodes is the most suitable model to study the intrinsic dynamics of DNA nanocages. Therefore, the following ENM results are derived from models based on three nodes.

### 2.2. Hinge Sites Predicted by the Slow Modes

**Figure 2:**
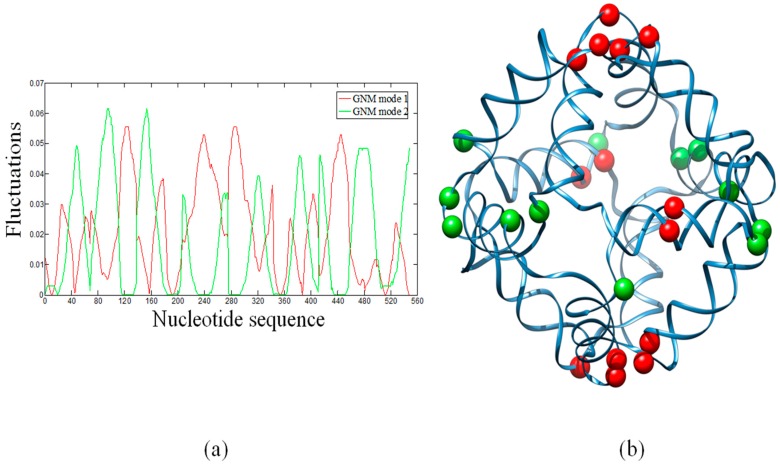
(**a**) Sequence-based fluctuations of the first and the second GNM modes of the DNA octahedron. Minima of the two modes correspond to hinge sites; (**b**) The distribution of hinge sites mapped to the three-dimensional structure. Red beads and green beads are hinges predicted by the first and the second GNM mode, respectively. The beads are mainly located over, or in the proximity, of the polyhedral vertices (i.e., the single strand thymidine bridges).

In ENMs, the slow modes represent the large-scale motions, which are usually relevant to the functions of biological systems. In particular, hinge sites in proteins are key residues that control global topologies and cooperative motions. In the global modes, hinge residues always have lowest mobilities and thus have the largest stability. Therefore, HingeProt [40] identifies hinge residues based on the minima of fluctuations of the two lowest GNM modes. Here, we have attempted to identify the distribution of hinge sites in the octahedral DNA cage. Figure 2a shows the fluctuation shapes of the first and second modes calculated by the GNM. Minima of the first GNM mode (red line) are displayed as red beads in the cartoon diagram (Figure 2b). Minima of the second GNM mode (Figure 2a, green line) are displayed as green beads (Figure 2b). The first mode indicates that hinge sites are mainly located at connecting regions (i.e., the single strand thymidine vertices) between the polyhedral edges (i.e., the double helices) (Figure S1) or their neighboring nucleotides, with the exception of a guanine and a thymidine that are located at the center of a DNA double helix. The second mode indicates that hinge sites are located around other connecting regions. GNM1 and GNM2 give different sets of hinge sites. Using the first two GNM modes, however, all the identified hinge sites are located around connecting regions (vertices) of the DNA octahedron.

Although many studies showed that the dynamics of DNA are sequence-dependent, our results show that hinge sites are simply imposed by the geometry of DNA cages, which confers a strong constraint to the mechanical/dynamical features. The slow modes analysis also shows that key nucleotides, controlling the stability of DNA cages, are located in the regions connecting edges and vertices. These data have validated by earlier MD and experimental results, which indicate that the stability and assembly yields are mainly influenced by the variation of single-stranded linker regions [14]. These hinge sites identify key nucleotides that may provide important clues for further assembly experiments and applications, including nanostructures designed to carry out opening/closing mechanisms.

### 2.3. Cross-Correlation Maps

**Figure 3:**
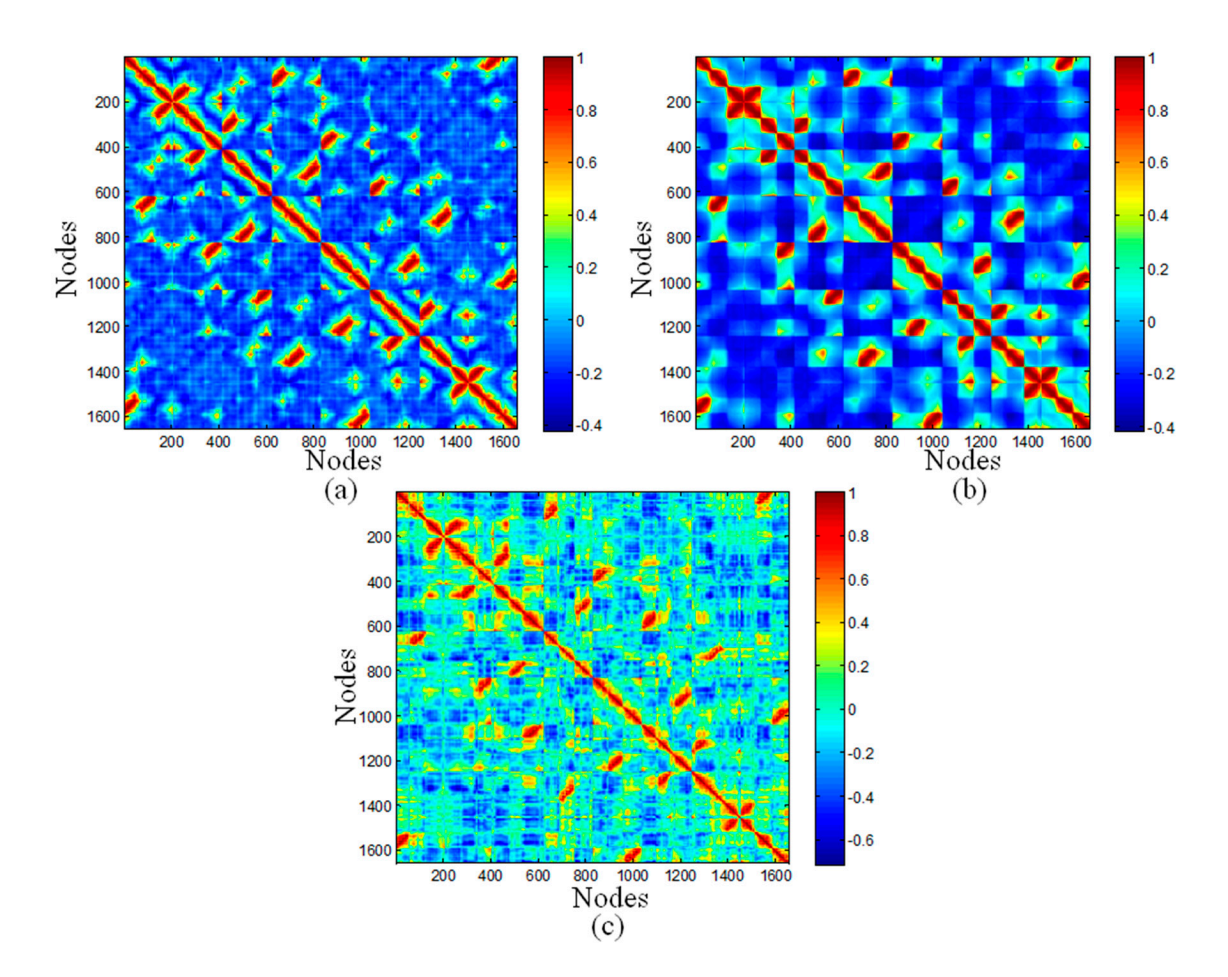
Correlation maps of the DNA octahedron predicted by: (**a**) the first 20 ANM modes; (**b**) the first 20 GNM modes; and (**c**) and the 50 ns MD simulation.

The top ranked Principal Component Analysis (PCA) and the lowest ENM modes often describe collective motions in which a number of nodes move in a concerted fashion. The dynamic cross-correlations maps for the DNA octahedron were obtained by both PCA based on MD simulation and ENM methods, which are represented in Figure 3. Since a small number of modes are enough to represent cross-correlation of nucleic acid systems [38], only 20 ANM and GNM modes have been used in the present calculation. Red regions in the cross-correlation map, with positive values, indicate the highly correlated motions, whereas the blue regions, with negative values, represent residues moving in anti-correlated motions. In Figure 3, the similarities between the three maps are striking. Twelve edges formed by DNA double strands have the highest correlations. In addition, two relatively weak positive correlations, including correlations between different vertices, as well as correlations between vertices and other edges, were observed in all three maps. Indeed, the correlation coefficients between MD and ANM, as well as GNM both equal to 0.71, pointing out the high similarity between MD and ENM cross-correlations.

The negative correlations have the most evident difference between different methods. The values in cross-correlation maps obtained by ANM, GNM and MD range from −0.4210 to 1, from −0.4166 to 1, and from −0.7152 to 1, respectively. Therefore, the highest value of negative correlation is found in the MD simulation, with the value of 0.7, while related smaller values are obtained in ANM and GNM, both with values of 0.4. The MD simulation showed higher negative correlations occurring between elements located far away. Accordingly, the whole structure of octahedral cage is contracting during the simulation, but this dynamical behavior cannot be detected by ANM and GNM.

### 2.4. Global Motions

In a series of previous works, PCA based on MD trajectories were used to detect the functional dynamics of DNA nanocages, but those calculations were carried out using the backbone phosphorus atoms (P) [13,14,15,16,17]. In this section, PCA analysis has been revised and the ENM motion analysis has been performed based on three nodes. The fractions of variance and collectivities of the first five modes of PCA, ANM and GNM are listed in Table 1. The top PCA modes show higher fractions of variance than the lowest ENM modes. PC1 and PC2 of the DNA octahedron account for 32% and 15% of the variance, respectively. Furthermore, the first 20 ANM and GNM modes both contribute more than half of the total variance (Table S3). The first five modes evaluated by three methods all correspond to highly collective motions. PCA and ANM were used to reveal the global motions of DNA octahedron, because the GNM cannot give the direction of the fluctuation.

**Figure 4:**
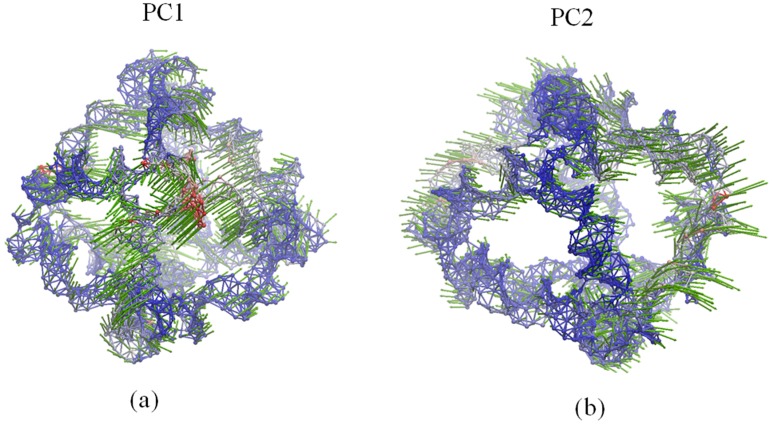
Dynamical motions predicted by: (**a**) PC1 and (**b**) PC2. Network representation of the DNA octahedron. Red and blue colors denote regions with high and low fluctuations, respectively, while arrows (green) denote the direction of the motions.

**Figure 5:**
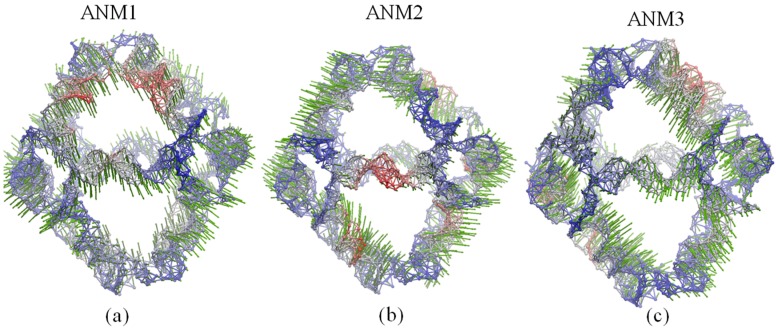
Global motions predicted by: (**a**) ANM1; (**b**) ANM2 and (**c**) ANM3. Red and blue colors denote regions with high and low fluctuations, respectively, while arrows (green) denote the direction of the motions.

In Figure 4, PC1 shows the rotational motions of DNA double helices and the opening/closing motion of one vertex hole, while PC2 indicates a combined motion due to the rotation of DNA double helices and the twisting/stretching motion of the whole polyhedron. Accordingly, as demonstrated in earlier MD simulations [13], the rotational motion of the DNA double helices represents the prominent dynamical activity of the DNA octahedron. As shown in Figure 5, the motions of the first three ANM modes indicate similar global motions consisting in the twisting and stretching of the whole octahedron. The rotational motion of the DNA double helices cannot be observed, but helices show different fluctuations (Videos S1–S3). In addition, the main global motion evidenced by the ANM analysis indicates a small contraction of the cage, which cannot be clearly detected by the cross-collection map of ANM.

### 2.5. Comparison of Anysotropic Network Model and Principal Component Analysis Modes

PCA modes can be compared with ANM modes to understand if the dynamics of the DNA octahedron are encoded by their native structures. The overlap map between the first ten PCA and ANM modes, displayed in Figure S4, indicates that a one-to-one correspondence between each mode in all systems is not present. The largest overlap was found for the PC2 and ANM2, showing the value of 0.58. These data indicates that the first two PCA modes can describe the large part of the DNA octahedral dynamics. We further investigate the relationship between these two PCA modes with the low-frequency ANM modes by calculating the overlap between the structural changes described by PC1 and PC2 and the first 20 ANM modes.

**Figure 6:**
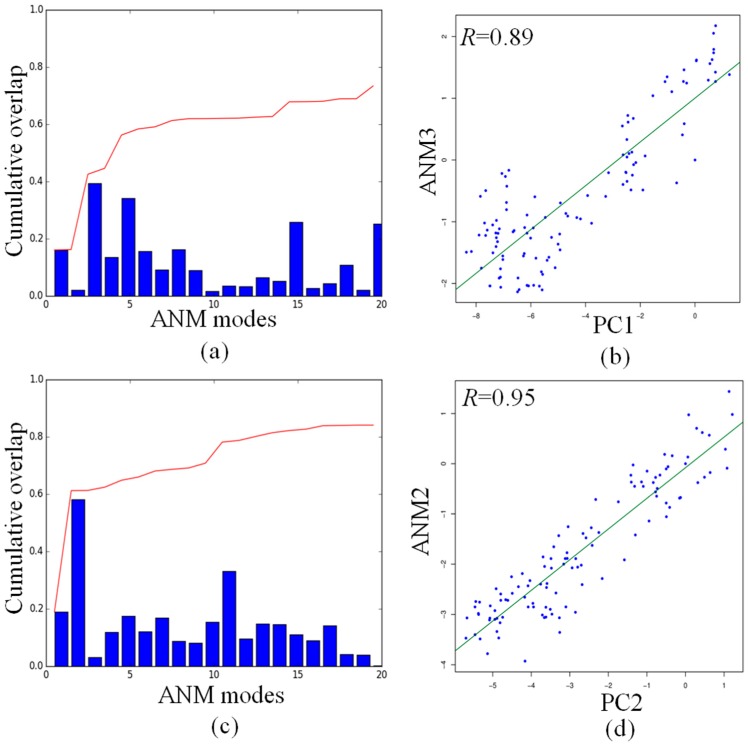
Comparisons of principal changes found in MD simulation and global motions predicted by ANM for the DNA octahedron: (**a**) the cumulative overlap between PC1 and the first ANM 20 modes; (**b**) cross-projection along PC1 and ANM3; (**c**) the cumulative overlap between PCs and the first ANM 20 modes; and (**d**) projection of the DNA octahedron along PC2 and ANM2.

As shown in Figure 6a, the cumulative overlap indicates that the first 20 ANM modes can explain about 70% of PC1, with the highest overlap of 0.39 between PC1 and ANM3. Figure 6c indicates that the cumulative overlap between the first 20 ANM modes and PC2 reaches the value of 0.8, while the highest overlap value of 0.58 has been found between PC2 and ANM2, as already shown in the overlap map in Figure S3. The projections of the ensemble of structures onto PC1 and ANM3 (Figure 6b), and PC2 and ANM2 (Figure 6d), yield the very high correlations values of 0.89 and 0.95, respectively. The consistent correlation between the subspace of conformations seen in PC1 and PC2 and those predicted by the single low-frequency ANM mode demonstrates that the dynamics of the DNA octahedron correspond, to a large extent, to those intrinsically encoded by the native contacts. The ANM calculation is only based on the initial structure from the Protein Data Bank, while MD simulation describe the process of how the DNA octahedron deformed to equilibrium. The high correlation between PC and ANM modes shows that the deformation of the DNA octahedron from initial structure to equilibrium is relatively small. It means that the DNA octahedron is intrinsically stable. This aspect further demonstrates, from another perspective, that the structure of DNA octahedron is intrinsically stable being a fully covalently closed molecule stabilized by hydrogen bonds and rings stacking in the double helix regions.

## 3. Discussion

### 3.1. Elastic Network Models: From Proteins to DNA

ENMs are dynamical approaches for the description of intrinsic dynamics of proteins near their native state. The main advantage over MD is that ENMs provide much quicker methods to study structures and functions of large protein complexes [41], still reproducing the collective motions and intrinsic flexibilities of proteins as accurately as all-atom models. Although the ENM methods were first proposed for proteins, their applications have been extended to nucleic acids, including DNA and RNA.

Isami and coworkers [42] applied the ENM to the analysis of nine long double-stranded DNA sequences, finding that the DNA fluctuations are sequence-dependent and that the inter-strand fluctuations show positive correlations with their nucleosome-forming ability. González and coworkers [43] indicated that ANM can provide useful insights into conformational dynamics of RNA CUG trinucleotide repeats (rCUG), including the global dynamics and fluctuations based on backbone and nucleobase of rCUG. Using ENM, Pinamonti and coworkers [44] have characterized the innate internal motions of several RNA secondary structures, which are related to biological functions. The predicted functional dynamics of RNA secondary structures have been verified by MD simulations and SHAPE experimental data. Zimmermann and Jernigan [45] have investigated the ability of using low-frequency normal modes from ENMs to predict dynamical motions for 16 ensembles of well-packed RNA structures, which were experimentally determined. Setny and Zacharias [46] have made their efforts to modify different parameters and developed some improved ENMs, which can be used to describe conformational changes and mobilities of DNA and RNA molecules.

### 3.2. Elastic Network Models for DNA: Form One Node to Three Nodes

For proteins, each residue is considered as one node always at Cα atom position. However, such coarse-graining level may be too extreme for the study of nucleic acids. To this aim, several ENMs for DNA and RNA molecules have been proposed. Among them, each nucleotide can be represented by one node, two or three nodes. Obviously, the ENM dynamics of DNA and RNA relies on the selection of the defined nodes.

In 1998, Bahar and Jernigan [47] first introduced a GNM with two nodes per nucleotide to study the conformational dynamics of a transfer RNA. In this way, each nucleotide can be represented as two nodes at P (phosphorous) and O4* (oxygen group) atoms, with the cut-offs for tRNA^Phe^ and tRNA^Asp^, and the intramolecular and intermolecular contacts of tRNA^Gln^ are 19 and 16 Å, respectively. Using the network model, some hinge sites of the free and synthetase-bound forms have been successfully identified. When considering tRNA–protein complexes, Wang and coworkers [48] modified the coarse-graining level and each nucleotide was represented as a node at P (phosphorous) atoms. The cut-off between P atoms was modified as well and changed to 24 Å. Although more local motions were neglected, the global motions can be accessed for much larger systems, even for 70S ribosome structure with more than 5000 nodes in the ENM. For the study of the conformational change between open and closed states of DNA-dependent polymerases, Delarue and Sanejouand [49] have used three nodes of P, C4* (sugar group) and C2 (base group) atoms to represent each nucleotide of the DNA molecule.

Despite these successes, using ENM to study the dynamics of DNA relevant nanocages remains elusive. The best parameterization for the DNA nanocage was obtained with an ENM in which each nucleotide is represented by three nodes, whose intrinsic flexibilities are mostly well agreed with the previous MD study.

### 3.3. Limitions of Elastic Network Models and Future Works

In comparison with MD simulations, the ENMs are coarse-grained methods that only describe the DNA dynamics around its equilibrium state using a harmonic potential function. Furthermore, ENMs consider neither solvent effects nor any type of interactions between atoms, which cannot describe some important local motions, derived from base pairs of DNA helices. For example, the geometric analysis of each double helix in DNA nanocages can be performed using MD, while ENMs cannot execute this kind of analysis. Moreover, the lack of capability of understanding the folding pathway is an additional limitation for both ENM and MD.

Despite of these limitations, the applications of ENMs in DNA nanocages still have great potential. We list some possible works in the future. (1) One major task in DNA nanotechnology is to self-assemble nanocages with increasing complexity. Although MD has been extensively used to describe the dynamics of a series of DNA nanocages, an atomic level description of the motions can be computationally prohibitive when these nanostructures become very large (i.e., edge widths of around 100 nm). In this case, ENMs calculations can easily provide significant motion information of large DNA nanocages; (2) DNA cages have a wide range of applications, such as drug delivery, which are facilitated by controlling the DNA cage between closed and opened states [50]. ENMs can detect which single mode gives a significant contribution to a conformational change, thus it is suitable for the understanding of complex molecular mechanisms such as drug encapsulation and release; (3) ENMs have biophysical meaning in the further molecular design [39]. DNA nanocages can be computationally designed by using different lengths of helical edges and connected vertices. ENM calculations can be easily used to predict their assembly properties and thermodynamics, by calculating general mechanical properties including intrinsic flexibilities and the size limits of motions. In addition, the minor change of hinge sites may affect the stabilities of DNA nanocages largely, suggesting important mutations for the experiment test. In summary, ENM approaches may provide helpful tools into the computational design of DNA nanostructures.

## 4. Materials and Methods

### 4.1. Computer Modeling and Molecular Dynamics Analysis

The molecular modeling of DNA nanocages, the equilibration and MD protocol were described in the previous work [27]. In this work, seven DNA nanocages with different geometries have been constructed. In the present work, we choose the most characterized DNA octahedron as the case study. In this octahedron, the DNA double-strand helices are composed by 18 base-pairs, while the single-strand linkers, connecting the double helices, are composed by five nucleotides. The total number of nucleotides in this octahedron is 552. The root-mean-square fluctuation (RMSF) and the correlation map based on MD simulation were calculated using the GROMACS 4.6.5 package [51].

The calculation of PCA is based on the construction of covariance matrixes *C* from the MD trajectory, whose elements are defined as

(1) $$\langle \left(R_{i}-\langle R_{i}\rangle \right)\cdot \left(R_{j}-\langle R_{j}\rangle \right)\rangle$$

where *R_i_* and *R_j_* denote position of network nodes and the brackets denote the average over trajectory frames. The diagonalization of covariance matrixes generates eigenvectors *u_k_* corresponding to PC modes.

### 4.2. Gaussian Network Model and Anisotropy Network Model

In both GNM and ANM, a DNA is considered as an elastic network, in which nodes are connected by harmonic springs with a force constant γ. The interaction potentials of the system in GNM and ANM are defined as

(2) $$V_{GNM}=\frac{\gamma }{2}\sum_{i,j}^{n}\left(R_{ij}-R_{ij}^{0}\right)\cdot (R_{ij}-R_{ij}^{0}),\;V_{ANM}=\frac{\gamma }{2}\sum_{i,j}^{n}{\left(|R_{ij}|-\left|R_{ij}^{0}\right|\right)}^{2}$$

where *n* is the number of springs, and ***R_ij_*^0^** and ***R_ij_*** are original and instantaneous distance vectors between node *i* and *j*. In our calculation, cut-offs for GNM based on one node, two nodes and three nodes are chosen at 20, 15 and 10 Å, respectively, whereas cut-offs for ANM based on one node, two nodes and three nodes are 30, 20 and 15 Å, respectively.

In GNM, the topology of the network is defined by the *n × n* Kirchhoff matrix **Г**, which is written as

(3) $$\Gamma_{ij}=\{\begin{array}{cc} -1 & i\neq j,\;R_{ij}\leq r_{c}\\ 0 & i\neq j,\;R_{ij}>r_{c}\\ -\sum_{i,i\neq j}\Gamma_{ij} & i=j \end{array}$$

where *R_ij_* is the distance between two nodes *i* and *j*. Square fluctuations are given by

(4) $$\langle {\left(\Delta R_{i}\right)}^{2}\rangle =\left(3kT/\gamma \right){\left[\Gamma^{-1}\right]}_{ii}\text{ and }\langle \Delta R_{i}\cdot \Delta R_{j}\rangle =\left(3kT/\gamma \right){\left[\Gamma^{-1}\right]}_{ij}$$

Thus, the cross-correlation can be calculated by

(5) $$C_{ij}=\frac{\langle \Delta R_{i}\cdot \Delta R_{j}\rangle }{{\left[\langle \Delta R_{i}^{2}\rangle \cdot \langle \Delta R_{j}^{2}\rangle \right]}^{1/2}}.$$

The motion of ANM modes are determined by Hessian matrix **H**, whose elements are given as

(6) $$H_{ij}=\left[\begin{array}{ccc} \frac{\partial^{2}V}{\partial x_{i}\partial x_{j}} & \frac{\partial^{2}V}{\partial x_{i}\partial y_{j}} & \frac{\partial^{2}V}{\partial x_{i}\partial z_{j}}\\ \frac{\partial^{2}V}{\partial y_{i}\partial x_{j}} & \frac{\partial^{2}V}{\partial y_{i}\partial y_{j}} & \frac{\partial^{2}V}{\partial y_{i}\partial z_{j}}\\ \frac{\partial^{2}V}{\partial z_{i}\partial x_{j}} & \frac{\partial^{2}V}{\partial z_{i}\partial y_{j}} & \frac{\partial^{2}V}{\partial z_{i}\partial z_{j}} \end{array}\right]$$

where *x_i_*, *y_i_* and *z_i_* represent the Cartesian components of nodes *i*. The ANM modes are corresponding to the eigenvectors *ν_l_* by the diagonalization of **H**.

### 4.3. Overlap of Modes

The overlap between ANM and PCA modes is used to evaluate the similarity between them, which is given by the dot product of the corresponding eigenvectors

(7) $$O(u_{k},v_{l})=u_{k}\cdot v_{l}$$

The cumulative overlap is used to quantify to which extent that a set of ANM soft modes can predict a PCA mode, thus it measures how well a subset of *m* ANM modes reproduces the *i*th PCA mode:

(8) CO(m)=[∑m=1m(O(uk,vl))2]12

### 4.4. Computational Detail

The PCA and ENM calculation were performed by using the ProDy software [52]. PCA and ANM modes were described by the percentage of total variance, *p*, and the collectivity degree, *k*, which was used as a measure of the number of atoms significantly affected by a given mode. A detailed description of MD simulation is listed in Supplementary Materials. For the comparison between ANM and MD, the coordinates of individual snapshots in the MD ensembles were saved every 500 ps, and a total of 100 frames were utilized for the computation of PCA modes.

## 5. Conclusions

In this paper, two Elastic Network Modes, namely GNM and ANM, have been used to study the intrinsic dynamics of a DNA octahedron. By comparing the fluctuations with the earlier MD simulation, the ENMs based on three nodes show the most consistent results. Using the first two normal modes of GNM, hinge sites of the DNA octahedron were predicted to be located at the connections between edges and vertices of the polyhedron. The cross-correlations, both calculated using the first 20 ANM and GNM modes, are in very good agreement with the values from the MD simulation. In addition, the exhibition of ANM global motions, compared with the PCA motions, indicates that low-frequency ANM modes are able to describe most of the dynamical properties. MD simulation is the classical method to describe equilibrium fluctuations caused by wiggling and jiggling. The well correspondence between ENM calculations and MD simulation suggest that DNA also has some well-defined intrinsic dynamics, encoded by the native fold [53]. We can expect that such kind of intrinsic dynamics plays a key role in mediating DNA–drug interactions. Although our work is limited to a DNA octahedron, we can expect that ENMs can be applied to explore the dynamic behavior and the conformational changes of other DNA cages with different geometries, providing useful insights for the molecular design in DNA nanotechnology.

## Figures and Tables

**Table 1:** Fraction of variance (*p*) and collectivity (*k*) of the first five Principal Component Analysis (PCA), ANM and GNM modes for the DNA octahedron.

**MD**	**PC1**	**PC2**	**PC3**	**PC4**	**PC5**
*p*	0.32	0.15	0.09	0.06	0.03
*k*	0.74	0.77	0.74	0.72	0.73
**ANM**	**ANM1**	**ANM2**	**ANM3**	**ANM4**	**ANM5**
*p*	0.06	0.05	0.05	0.04	0.04
*k*	0.81	0.79	0.82	0.73	0.81
**GNM**	**GNM1**	**GNM2**	**GNM3**	**GNM4**	**GNM5**
*p*	0.10	0.09	0.07	0.04	0.04
*k*	0.66	0.54	0.56	0.65	0.49

## Supplementary Materials

Supplementary materials can be accessed at: http://www.mdpi.com/1420-3049/22/1/145/s1.
