# Supplementary material for: Intrinsic Dynamics Analysis of a DNA Octahedron by Elastic Network Model
Source: Molecules. 2017 Jan 16;22(1):145. doi: 10.3390/molecules22010145 (PMC6155889; doi:10.3390/molecules22010145)
Supplement: Supplementary file 1 [file molecules-22-00145-s001.zip › molecules-166283-supplementary/molecules-166283-supplementary .pdf]

Supplementary Materials: Intrinsic Dynamics Analysis of a DNA Octahedron by Elastic Network Model

Guang Hu, Lei He, Federico Iacovelli and Mattia Falconi

1. Molecular Dynamics Simulation of the Octahedral DNA Nanocage

1.1. Nanocage Model Preparation for Molecular Dynamics Simulation

The system topology and the coordinates, used as input for the NAMD 2.10 MD package [1], have been obtained through the AmberTools14 tLeap [2] module, parameterizing the structure through the AMBERFF14SB force-field with the parmbsc1 correction [3]. The structure has been immersed in a suitable box (Table S1), filled with TIP3P water molecules [4], imposing a minimum distance between the solute and the box of 14 Å, whereas the charges have been neutralized adding Mg^{2+} counter-ions to the solvated systems [5] (Table S1) in favorable positions, as implemented in the AMBER 14 program [2].

Table S1. Parameters for the box of the DNA octahedral nanocages

**Octahedral DNA Nanocage**	
Box size in Å (X,Y,Z)	145,145,145
Total number of atoms	207,762
DNA atoms	17,544
Number of nucleotides	552
Water molecules (TIP3P)	63,314
Number of ions (Mg^{2+})	276

1.2. Equilibration and Molecular Dynamics Protocol

A minimization run has been performed for 5000 steps using the steepest descent algorithm, imposing harmonic constraint of 50 kcal·mol^{-1}·Å$^{-2,}$ to remove any unfavorable interaction and to prevent irreversible Mg^{2+} binding to DNA. The system has been gradually heated in the canonical ensemble (NVT) from 0 K to 300 K over a period of 500 ps using Langevin thermostat [6] with coupling coefficient of 1.0 ps and a weak constraint of 15 kcal·mol^{-1}·Å^{-2} on nucleotides. In the last equilibration step, the system was subjected to an equilibrium simulation for 500 ps removing all constraints. The optimized system has been then simulated using the isobaric-isothermal ensemble (NPT) for 50 ns, using periodic boundary conditions, a 2.0 fs time-step, a cut-off of 9 Å for the evaluation of short-range non-bonded interactions and the PME method [7] for the long-range electrostatic interactions. The SHAKE algorithm has been used to constrain covalent bonds involving hydrogen atoms [8]. Temperature has been fixed at 300 K using the Langevin dynamics [6], while pressure has been held constant at 1 atm through the Langevin piston method [9]. The atomic positions have been saved every 500 steps (1.0 ps) for the analyses. As observed in previous MD simulations 50 ns is a time sufficient to sample the conformational properties of the octahedral DNA nanocage. The simulation has been entirely performed using 256 nodes for a total of 4096 cores of the FERMI HPC Cluster hosted by CINECA, Bologna, Italy.

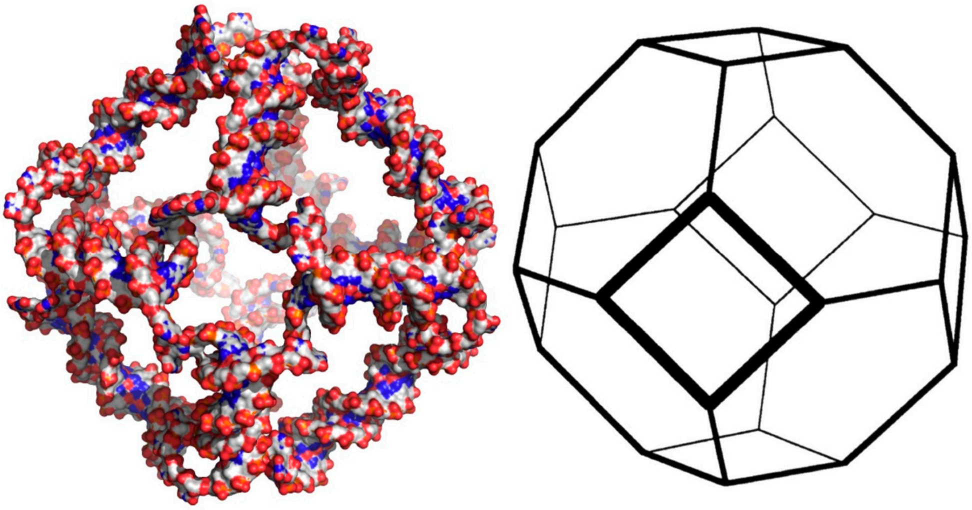

Figure S1. Atomistic (**left**); and schematic (**right**) representations of the octahedral DNA nanocage shown with the same orientation. In the atomistic representation, the surface of the nanocage is colored by atom type. In the schematic image, the squares represent the vertices of the polyhedron that are composed by DNA single strands. The remaining sides building up the hexagons indicate the polyhedron edges and are composed by DNA double helices.

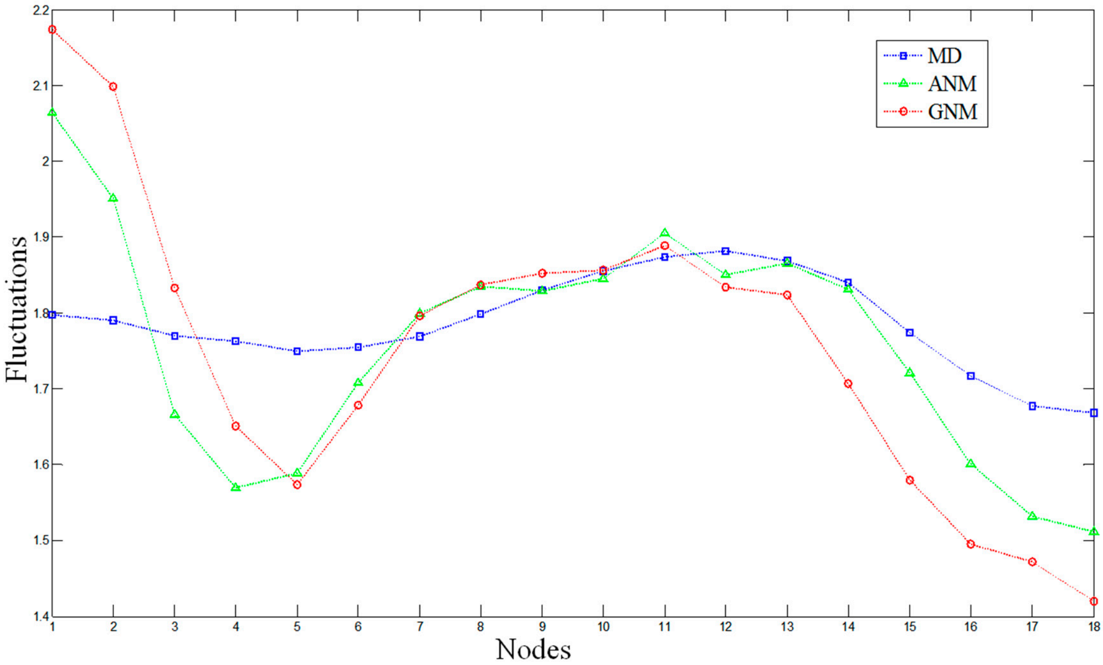

Figure S2. The RMS fluctuations of the DNA octahedron derived by MD (the blue line), ANM (the green line), and GNM (the red line). The RMSFs based on ANM and GNM are calculated with each nucleotide represented by one node. Average fluctuations for only one strand composing the double helices are plotted.

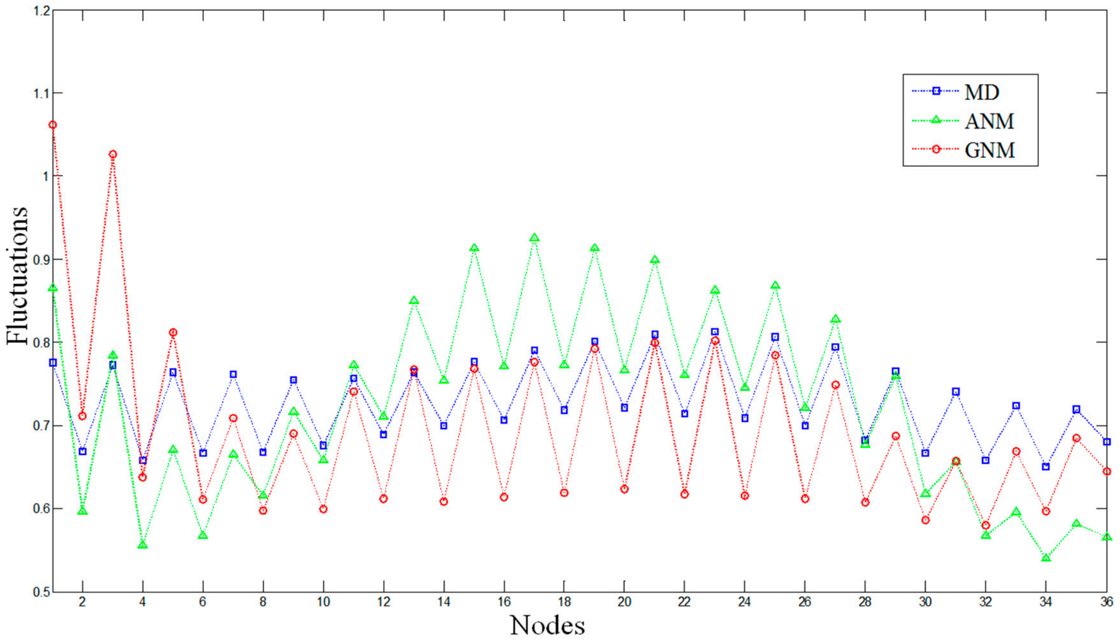

Figure S3. The RMS fluctuations of the DNA octahedron derived by MD (the blue line), ANM (the green line), and GNM (the red line). The RMSFs based on ANM and GNM are calculated with each nucleotide represented by two nodes. Average fluctuations for only one strand composing the double helices are plotted.

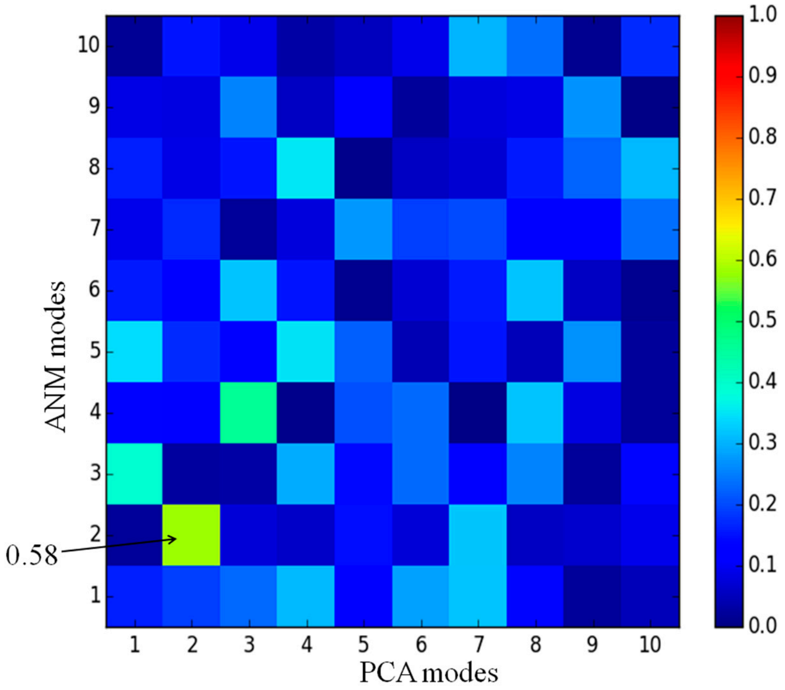

Figure S4. The overlap maps between the first ten PCA and ANM modes of the DNA octahedron, with the PC2 exhibiting the highest overlap (0.58) with ANM2.

Table S2. The eight DNA oligonucleotides composing the octahedral DNA nanocage. The annealing regions are shown in color and non-annealing spacer regions in black. The black thymidines indicate the single DNA strands connecting the double helices and defining the vertices of the polyhedron. The different colors show the complementary regions that generate the double helices defining the polyhedron edges.

Oligo	Octahedral DNA Nanocage
1	GCCACCAGGTTTTCGATGTCTAAGCTGACCGTTTTGGACCGTGATTCCATGACTTTTCTTAGAGTT
2	TGGCTACAGTTTTCGGTCAGCTTAGACATCGTTTTGAATCCTATGCTCGGACGTTTTGGCTCACAT
3	TCACGGTCCTTTTCTATCCGATCGAGGCATGTTTTCATACTGAGAGCGTTCCGTTTTGTCATGGAA
4	CAGATACGCTTTTCATGCCTCGATCGGATAGTTTTCTGTAGCCAATGTGAGCCTTTTGTCGCAGTT
5	CTCAGTATGTTTTCGGTTACGGTACAATGCCTTTTCGCAAGACGTTAGTGTCCTTTTCGGAACGCT
6	GGTGTATCGTTTTGGCATTGTACCGTAACCGTTTTGCGTATCTGAACTGCGACTTTTCCACCGAAT
7	CGTCTTGCGTTTTGTATGACGCAGCACTTGCTTTTCCTGGTGGCAACTCTAAGTTTTGGACACTAA
8	ATAGGATTCTTTTGCAAGTGCTGCGTCATACTTTTCGATACACCATTCGGTGGTTTTCGTCCGAGC

Table S3. Fraction of variance (p) of the first 20 ANM and GNM modes for the DNA octahedron.

Methods	Fraction of Variance (p) for the First 20 Modes	Sum of Fraction of Variance
ANM	0.06, 0.051, 0.048, 0.04, 0.039, 0.034, 0.028, 0.028, 0.026, 0.023, 0.021, 0.021, 0.015, 0.013, 0.013, 0.011, 0.011, 0.009, 0.009, 0.008	0.507
GNM	0.097, 0.089, 0.072, 0.041, 0.038, 0.033, 0.028, 0.023, 0.022, 0.02, 0.015, 0.014, 0.014, 0.013, 0.013, 0.012, 0.011, 0.011, 0.009, 0.009	0.584

References

1. Phillips, J.C.; Braun, R.; Wang, W.; Gumbart, J.; Tajkhorshid, E.; Villa, E.; Chipot, C.; Skeel, R.D.; Kalé, L.; Schulten, K. Scalable Molecular Dynamics with NAMD. *J. Comput. Chem.* **2005**, *26*, 1781–1802.
2. Salomon-Ferrer, R.; Case, D.A.; Walker, R.C. An Overview of the Amber Biomolecular Simulation Package. Wiley Interdiscip. Rev. Comput. *Mol. Sci.* **2013**, *3*, 198–210.
3. Ivani, I.; Dans, P.D.; Noy, A.; Pérez, A.; Faustino, I.; Hospital, A.; Walther, J.; Andrio, P.; Goñi, R.; Balaceanu, A.; et al. Parmbsc1: A Refined Force Field for DNA Simulations. *Nat. Methods* **2016**, *13*, 55–58.
4. Jorgensen, W.L.; Chandrasekhar, J.; Madura, J.D.; Impey, R.W.; Klein, M.L. Comparison of Simple Potential Functions for Simulating Liquid Water. *J. Chem. Phys.* **1983**, *79*, 926.
5. Li, P.; Roberts, B.P.; Chakravorty, D.K.; Merz, K.M. Rational Design of Particle Mesh Ewald Compatible Lennard-Jones Parameters for +2 Metal Cations in Explicit Solvent. *J. Chem. Theory Comput.* **2013**, *9*, 2733–2748.
6. Nosé, S. A Unified Formulation of the Constant Temperature Molecular Dynamics Methods. *J. Chem. Phys.* **1984**, *81*, 511.
7. Darden, T.; York, D.; Pedersen, L. Particle Mesh Ewald: An N·log(N) Method for Ewald Sums in Large Systems. *J. Chem. Phys.* **1993**, *98*, 10089.
8. Ryckaert, J.; Ciccotti, G.; Berendsen, H. Numerical Integration of the Cartesian Equations of Motion of a System with Constraints: Molecular Dynamics of N-Alkanes. *J. Comput. Phys.* **1977**, *23*, 327–341.
9. Feller, S.E.; Zhang, Y.; Pastor, R.W.; Brooks, B.R. Constant Pressure Molecular Dynamics Simulation: The Langevin Piston Method. *J. Chem. Phys.* **1995**, *103*, 4613.
